# A tribute to Sidney Altman, one of the architects of modern RNA biology

**DOI:** 10.1016/j.jbc.2024.107364

**Published:** 2024-05-11

**Authors:** Venkat Gopalan, Leif A. Kirsebom

**Affiliations:** 1 Department of Chemistry & Biochemistry, Center for RNA Biology, The Ohio State University, Columbus, Ohio, USA; 2 Department of Cell and Molecular Biology, Uppsala University, Uppsala, Sweden

**Keywords:** Sidney Altman, discovery of RNA catalysis, endonucleases

## Abstract

This special issue of *JBC* pays tribute to Sidney Altman, whose discovery of a catalytic role for RNA, a breakthrough made independently by Thomas Cech, overturned the long-held dogma that only proteins can serve as catalysts in biological systems. The discovery of RNA catalysis galvanized biologists to think expansively in new directions and has given rise to a remarkable RNAissance in science and medicine. The collection of articles begins with the story of the discovery of RNase P and builds up to the emerging picture of an unexpectedly vast repertoire of RNase P variants in the three domains of life, including insights derived from recent high-resolution structures on how RNAs, ribonucleoproteins, or protein scaffolds can be used variably to generate an active site for catalyzing the same RNA processing reaction. The series of articles ends with a discussion of more recently discovered endonucleases (Argonautes, Cas), whose parallels with RNase P underscore recurring themes in diverse biological contexts.

“Science, like the Mississippi, begins in a tiny rivulet in the distant forest. Gradually other streams swell its volume. And the roaring river that bursts the dikes is formed from countless sources.” *Abraham Flexner*.

We celebrate the research contributions of Sidney Altman, one of the architects of modern RNA biology, an era that exemplifies Flexner’s delightful description of science (1). Trained as a physicist, biophysicist, and molecular biologist, Altman made sense of the unexpected and used that knowledge innovatively to contribute to RNA biochemistry and biotechnology. While the history of RNA catalysis by RNase P (2, 3, 4) and Altman’s excellence as a scientist and mentor have been described elsewhere (5, 6), this commemorative issue is a tribute to how Altman’s quest to understand a seemingly commonplace biological reaction opened a new chapter in biocatalysis and molecular recognition, while also spawning novel RNA-based regulatory agents and therapeutics. Some of the salient points in each of the six articles in this collection are summarized below (7, 8, 9, 10, 11, 12).

The history of RNase P, the tRNA-processing enzyme that was the focus of Altman’s research career, tracks the chronology of contemporary developments in molecular biology. Kirsebom *et al.* (7) begin the story by describing how Sydney Brenner’s elegant genetic experiments with the amber suppressor tRNA^Tyr^Su3 paved the way for Altman’s identification of a precursor tRNA (pre-tRNA) with a long 5′ leader, motivating and facilitating the search for the tRNA 5′-maturation enzyme. When bacterial RNase P was finally isolated and found to consist of an RNA molecule and a protein subunit, the availability of *in vitro* transcription systems allowed the unambiguous demonstration that the RNA moiety of RNase P was, itself, the catalyst! Altman shared with Thomas Cech the 1989 Nobel Prize in Chemistry for their independent discoveries of RNA catalysis (Fig. 1).

**Figure 1:**
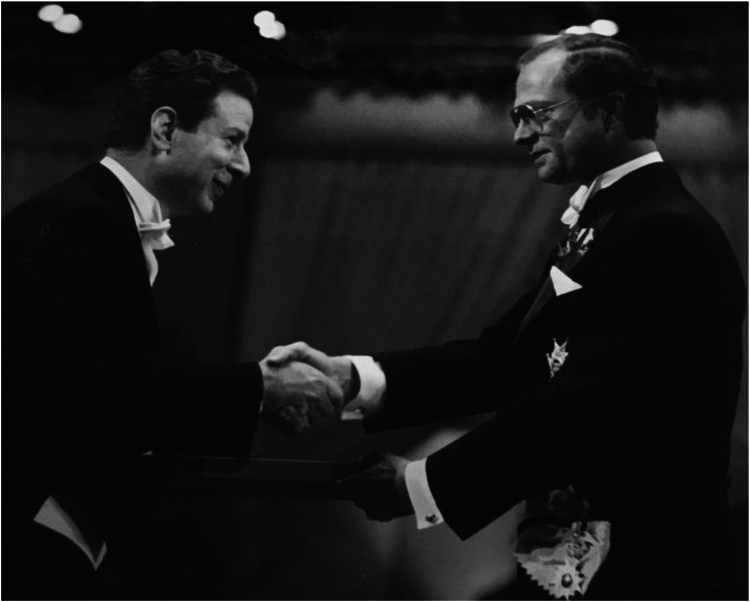
**Sidney Altman receiving the Nobel Prize in Chemistry from King Carl XVI Gustaf of Sweden in 1989.** Photograph courtesy of Ann M. Altman.

Subsequent deletion mutagenesis and biochemical studies demonstrated that the RNA of RNase P is constructed from smaller functional modules, that it exercises “induced fit” for catalysis, and that it can use Mg^2+^ ions for the site-specific cleavage of pre-tRNA. Although these striking parallels with protein enzymes may appear remarkable at first glance, all biocatalysts can, perhaps, be expected to leverage similar bond-breaking strategies. Paring down the pre-tRNA to smaller model substrates, in conjunction with the development of methods for the chemical synthesis of short RNAs, allowed the incorporation of deoxynucleotides and unnatural nucleobases at different positions in the substrate. As summarized by Kirsebom *et al.* (7), this capability helped determine how specific structural elements and functional groups in the pre-tRNA affected RNase P-mediated catalysis. Moreover, the finding that an RNase P substrate could be assembled from two RNAs, which when hydrogen-bonded together resemble the natural pre-tRNA, inspired Altman *et al.* to propose and validate the novel idea that a target RNA-specific guide RNA and endogenous RNase P could be used to inactivate any cellular RNA. This method, which has now been used for different applications (7), presaged the development of other RNA-based knockdown tools (*e.g.*, RNAi).

All multi-substrate RNA-processing enzymes face the challenge of accommodating structural variations in their suite of cognate substrates and discriminating effectively against non-cognate cellular substrates. Harris *et al.* explain why RNase P, which catalyzes 5′ maturation of all pre-tRNA isoacceptors (mono-/poly-cistronic contexts) and some other non-coding RNAs (*e.g.*, 4.5S RNA), provides an excellent model system with which to delineate and define the factors that contribute to this specificity landscape (8). To gain insights into the substrate specificity of bacterial RNase P, Harris *et al.* used an approach called High Throughput Sequencing-Kinetics (HTS-Kin), which combines RNA-processing assays and Illumina sequencing, and then integrated their HTS-Kin findings with the results of cryogenic-electron microscopic (cryo-EM) studies of enzyme-substrate (ES) complexes. The HTS-Kin studies established the relative rate constants for large populations of randomized pre-tRNAs and provided new insights and/or reaffirmed previously established positive and negative determinants (*e.g.*, pairing of the 5′ leader and the 3′ ACCA results in a longer acceptor stem and is an anti-determinant).

The cryo-EM structure of bacterial RNase P bound to a pre-tRNA with an extended acceptor stem revealed that the protein and RNA subunits of bacterial RNase P act collaboratively as a wedge to separate the 5′ leader and the 3′ ACCA (8); in this example, a dynamic ensemble of bacterial RNase P and pre-tRNA conformations (rather than the pre-organized structures in archaeal/eukaryotic counterparts; see ref. (9)) helps generate productive ES complexes. Specifically, local conformational flexibility was postulated to be mandatory for the cross-domain reorganization and accommodation of substrates that lacked key recognition elements. However, unwinding of substrates with extended acceptor stems must incur energetic penalties, a feature that Chamberlain *et al.* (8) then consider, in the context of a kinetic framework, as the mechanistic basis for substrate specificity. They also discuss how insights into molecular recognition by RNase P should help realize the promise of bacterial RNase P as a target for new antibiotics.

Studies on bacterial RNase P predictably prompted the characterization of eukaryotic and archaeal relatives, an objective greatly aided by the arrival of tandem mass spectrometry (MS/MS) proteomics (mid-1990s). Unlike the one RNA–one protein RNase P holoenzyme in bacteria, eukaryotic RNase P was found to contain a single RNA but as many as ten proteins; the archaeal cousin is intermediate in terms of complexity, having one RNA and up to five proteins (that have a shared origin with eukaryotic counterparts). However, the RNA in these more complicated RNase P ribonucleoproteins (RNPs) remains the catalytic subunit. Lei *et al.* describe the results of cryo-EM analysis of the structures of yeast, human, and archaeal RNase P that reveal tightly interwoven RNA and protein subunits, a feature that reflects their co-evolution (9). The structures of these variants, when bound to pre-tRNA/tRNA, also highlight the fact that the structural diversity of bacterial, archaeal, and eukaryotic RNase P belies the uniformity of their mechanisms of action. All variants examined exploit a conserved active-site architecture to (i) position two metal ions that are obligatory for catalysis, and (ii) form a double-anchor ruler to measure the coaxially stacked acceptor-T stem before licensing accurate cleavage of the pre-tRNA. In view of these thematic similarities, the way in which the biological function of the catalytic RNA in RNase P is tweaked by a variable suite of associated protein cofactors remains an unresolved mystery. Key insights regarding the origins of biological catalytic diversity have emerged from comparisons of the cryo-EM structures of yeast RNase P and RNase MRP (mitochondrial RNA processing), which contain distinct RNAs but, nevertheless, share eight protein subunits. Surprisingly, two of these common proteins exhibit different conformations in RNases P and MRP, an indication of how RNAs and proteins mutually influence their functional repertoires and, most probably, shaped the evolution of RNPs.

If Altman’s findings challenged an orthodoxy, they unintentionally gave rise to a new belief, namely, that all RNase Ps must contain a catalytic RNA. This idea was disproved by the early work of Peter Gegenheimer followed by the *tour de force* biochemical studies of Walter Rossmanith *et al.*. The existence of RNA-free, protein-only RNase P (PRORP) provided a lesson in the dangers of being confined to received wisdom. As described by Rossmanith, Giegé and Hartmann (10), eukaryotic PRORPs (like the evolutionarily unrelated RNP versions) come in different flavors: some are embedded in multi-enzyme assemblies (*e.g.*, human mitochondrial RNase P) and others function as free-standing, single polypeptides (*e.g.*, the nuclear and organellar variants in *Chlamydomonas*) (10). The subsequent discovery of a bacterial/archaeal enzyme that was named Homolog of *Aquifex* RNase P (HARP) indicated that the protein-only enzyme evolved independently, twice, to generate the same metallonuclease fold. Since HARP is present in some bacteria/archaea that also have the RNP form of RNase P, which appears to be the main player in tRNA 5′ processing, HARP might have other functions, including some related to its surprisingly large dodecameric assemblies. Various biochemical/biophysical studies of the PRORP monomer (full-length or truncated) either alone or bound to a pre-tRNA established that the RNA- and protein-based forms of RNase P both position two metal ions appropriately for site-specific hydrolysis and “measure” the distance between the tRNA elbow and the cleavage site. This thematic overlap between RNA- and protein-based active sites, both of which have evolved to cleave a specific phosphodiester linkage in pre-tRNAs, is a beautiful example of convergent evolution.

MicroRNAs (miRNAs; ∼22 nt) bind to the Argonaute (AGO) family of proteins to generate RNA-induced silencing complexes (RISCs). The resident miRNA in each RISC serves as a guide for the binding of a complementary target mRNA, eliciting either target cleavage (slicer activity) or translational repression. Nakanishi reviews recently published high-resolution structures of prokaryotic and eukaryotic AGOs and underscores similarities and differences to help us better understand the functions of and evolutionary relationships among them (11). For example, despite the presence of a common pseudo-catalytic tetrad (DEDH or DEDD), not all AGOs are active. Structures of various apo-/holo-RISC complexes reveal how propagation of the pairing of the guide miRNA and the target RNA elicits nuanced molecular rearrangements which, in turn, enable precise positioning of the catalytic tetrad and the two Mg^2+^ ions necessary for cleavage of the target RNA. The molecular gymnastics involving protein/RNA and Mg^2+^ ions provide exquisitely detailed information about how AGOs catalyze site-specific endonucleolytic cleavage of a phosphodiester linkage even without recognizing any tertiary structural elements, and why certain AGOs (*e.g.*, human AGO4) that possess the catalytic tetrad but lack key residues for firm Mg^2+^-chelation are inactive. Nakanishi also lists questions for profitable future research including: Why do metazoans favor slicer-independent translational repression over cleavage and how did proteomic complexity influence this scenario? What physiological cues and stressors shape the variable activation of different AGOs by RNAs of differing lengths? Answers to these questions are eagerly anticipated, given the tremendous interest in programming AGOs with exogenous guide RNAs to alter gene expression and to treat human diseases.

In all life forms, innate immunity depends on the ability to discriminate self from non-self. The CRISPR-Cas RNP enzyme catalyzes a targeted break in “invading” DNA/RNA and functions primarily to protect prokaryotes against mobile genetic elements. The use of RNAs that have sequence complementarity to the invading nucleic acid is at the heart of all CRISPR-Cas-based adaptive immune systems, which are now categorized as Classes 1 and 2 (each with three types). Based on a comprehensive survey of the results of many bioinformatic, biochemical, and structural studies, Ganguly *et al.* (12) summarize the differences in and similarities among the different CRISPR-Cas systems with respect to surveillance, target recognition, and the catalytic mechanisms used for DNA/RNA cleavage. The use of a multi-subunit complex (Class 1) *versus* a single multi-domain protein (Class 2) gives rise to different structural platforms, but these diverse structures share the common objective of ensuring that the molecular scissors have specificity determinants that primarily recognize non-self-nucleic acids. Similar to AGOs, the CRISPR-Cas systems use the “seed” feature of the RNA as a guide (11, 12). Site-specific cleavage is accomplished by divalent cation-dependent or -independent mechanisms, revealing plasticity with respect to general acid-base catalysis for completion of the same task (in a manner mirroring different ribozymes). To successfully disarm phages and to trigger cell death/dormancy, the CRISPR-Cas systems also activate second messenger synthesis that in turn regulates different cellular processes (*e.g.*, transcription, non-specific nucleic acid cleavage). The signaling events and ancillary proteins that trigger this intricate crosstalk remain to be elucidated. Ganguly *et al.* (12) suggest other directions for further fruitful research: The need for a better understanding of subtype diversity and functional gains; discovery and characterization of eukaryotic versions (*e.g.*, Fanzor proteins); and the application of these diverse systems to customized gene editing in both laboratory and clinical settings.

Together, the six reviews in this issue showcase the roles of RNAs as principal players in a wide variety of catalysts, a feature that some may argue reflects the RNA World heritage of all life forms. Thematic similarities abound in the mechanisms of structurally dissimilar enzymes, for example, their shared ability to (i) act as molecular calipers that can “size” substrates, (ii) augment with metal ions the catalytic potential of amino acids/nucleobases in protein/RNA enzymes, and (iii) minimize off-target cleavages by effecting obligatory conformational changes, as well as exploiting multiple recognition-determinants. It is difficult to parse how, during evolution, both chance and necessity might have fashioned the individual inventories of RNase P, AGO, and CRISPR/Cas variants. Subsumed within this larger story, however, are implications as to the way in which the ancient RNA World might have gradually transitioned to yield the extant RNP/protein-dominated state.

By demonstrating that the biological roles of RNAs are not formulaic and that RNAs can be enzymes, Altman and Cech opened new avenues of RNA research unimpeded by the restraint of longstanding disciplinary beliefs. Like the perils of monocultures in agriculture, their discovery also showed why scientific monocultures can undermine efforts to build scientific knowledge and to enhance our understanding of the natural world. As illustrated in this special memorial issue, Altman’s inspiring research attests to the payoffs for the human mind and spirit when curiosity is pursued solely for its own sake.

## Conflict of interest

The authors declare that they have no conflicts of interest with the contents of this article
